# Supplementary material for: Serum galectins as potential biomarkers of inflammatory bowel diseases
Source: PLoS One. 2020 Jan 13;15(1):e0227306. doi: 10.1371/journal.pone.0227306 (PMC6957193; doi:10.1371/journal.pone.0227306)
Supplement: S1 File — (DOCX) [file pone.0227306.s001.docx]

**Table S1. Galectin-1 expression according to disease duration:**

| Duration | Controls  (n=40) | Crohn’s disease (n=97) | Ulcerative colitis (n=71) | All IBD  (n=168) |
| --- | --- | --- | --- | --- |
| None |  |  |  |  |
| N | 15 |  |  |  |
| Mean (SD), ng/ml | 6.2 (14.2) |  |  |  |
| Median (range), ng/ml | 0.7 (0, 48.1) |  |  |  |
|  |  |  |  |  |
| <1 year |  |  |  |  |
| N |  | 6 | 7 | 13 |
| Mean (SD), ng/ml |  | 7.8 (7.4) | 54.6 (118.4) | 33.0 (87.3) |
| Median (range), ng/ml |  | 5.9 (0.8, 22.2) | 10.1 (4.2, 322.9) | 7.5 (0.8, 322.9) |
|  |  |  |  |  |
| 1-5 years |  |  |  |  |
| N |  | 12 | 17 | 29 |
| Mean (SD), ng/ml |  | 9.9 (8.0) | 39.6 (107.5) | 27.3 (82.7) |
| Median (range), ng/ml |  | 8.4 (1.0, 26.8) | 14.5 (0.7, 454.8) | 11.8 (0.7, 454.8) |
|  |  |  |  |  |
| 5-10 years |  |  |  |  |
| N |  | 17 | 35 | 52 |
| Mean (SD), ng/ml |  | 83.7 (313.5) | 58.5 (183.9) | 66.7 (231.3) |
| Median (range), ng/ml |  | 8.6 (0.3, 1300) | 10.9 (0, 815.9) | 9.2 (0, 1300) |
|  |  |  |  |  |
| >10 years |  |  |  |  |
| N |  | 43 | 0 | 43 |
| Mean (SD), ng/ml |  | 43.1 (183.1) |  | 43.1 (183.1) |
| Median (range), ng/ml |  | 7.7 (0.3, 1210.1) |  | 7.7 (0.3, 1210.1) |
|  |  |  |  |  |
| Missing |  |  |  |  |
| N |  | 3 | 2 | 5 |
| Mean (SD), ng/ml |  | 6.5 (7.8) | 26.1 (32.2) | 14.4 (20.1) |
| Median (range), ng/ml |  | 4.0 (0.4, 15.3) | 26.1 (3.4, 48.9) | 4.0 (0.4, 48.9) |
|  |  |  |  |  |

**Table S2. Galectin-2 expression according to disease duration:**

| Duration | Controls  (n=40) | Crohn’s disease (n=97) | Ulcerative colitis (n=71) | All IBD  (n=168) |
| --- | --- | --- | --- | --- |
| None |  |  |  |  |
| N | 20 |  |  |  |
| Mean (SD), ng/ml | 177.7 (616.5) |  |  |  |
| Median (range), ng/ml | 0.6 (0, 2669.3) |  |  |  |
|  |  |  |  |  |
| <1 year |  |  |  |  |
| N |  | 6 | 7 | 13 |
| Mean (SD), ng/ml |  | 1154.4 (2216.3) | 102.7 (270.0) | 588.1 (1543.0) |
| Median (range), ng/ml |  | 19.2 (0, 5545.4) | 0 (0, 714.9) | 0 (0, 5545.4) |
|  |  |  |  |  |
| 1-5 years |  |  |  |  |
| N |  | 13 | 20 | 33 |
| Mean (SD), ng/ml |  | 4740.4 (15558.2) | 39.2 (129.7) | 1891.2 (9809.3) |
| Median (range), ng/ml |  | 1.4 (0, 56371.4) | 0 (0, 571.5) | 0 (0, 56371.4) |
|  |  |  |  |  |
| 5-10 years |  |  |  |  |
| N |  | 18 | 37 | 55 |
| Mean (SD), ng/ml |  | 247.0 (780.0) | 4643.0 (16983.3) | 3204.3 (14029.1) |
| Median (range), ng/ml |  | 0 (0, 3200.7) | 0.1 (0, 99546.4) | 0.1 (0, 99546.4) |
|  |  |  |  |  |
| >10 years |  |  |  |  |
| N |  | 46 | 0 | 46 |
| Mean (SD), ng/ml |  | 433.2 (2040.8) |  | 433.2 (2040.8) |
| Median (range), ng/ml |  | 0.1 (0, 13733.9) |  | 0.1 (0, 13733.9) |
|  |  |  |  |  |
| Missing |  |  |  |  |
| N |  | 4 | 2 | 6 |
| Mean (SD), ng/ml |  | 2.2 (4.5) | 98.2 (138.8) | 34.2 (79.5) |
| Median (range), ng/ml |  | 0 (0, 9.0) | 98.2 (0, 196.4) | 0 (0, 196.4) |

**Table S3. Galectin-3 expression according to disease duration:**

| Duration | Controls  (n=40) | Crohn’s disease (n=97) | Ulcerative colitis (n=71) | All IBD  (n=168) |
| --- | --- | --- | --- | --- |
| None |  |  |  |  |
| N | 23 |  |  |  |
| Mean (SD), ng/ml | 22.0 (22.1) |  |  |  |
| Median (range), ng/ml | 14.9 (0, 96.4) |  |  |  |
|  |  |  |  |  |
| <1 year |  |  |  |  |
| N |  | 8 | 7 | 15 |
| Mean (SD), ng/ml |  | 48.2 (34.5) | 84.4 (99.1) | 65.1 (71.8) |
| Median (range), ng/ml |  | 45.4 (3.0, 110.2) | 29.4 (13.8, 276.2) | 44.3 (3.0, 276.2) |
|  |  |  |  |  |
| 1-5 years |  |  |  |  |
| N |  | 14 | 21 | 35 |
| Mean (SD), ng/ml |  | 55.2 (72.3) | 35.4 (26.0) | 43.3 (49.9) |
| Median (range), ng/ml |  | 36.2 (0, 282.1) | 27.5 (0.7, 98.6) | 29.2 (0, 282.1) |
|  |  |  |  |  |
| 5-10 years |  |  |  |  |
| N |  | 18 | 40 | 58 |
| Mean (SD), ng/ml |  | 51.3 (28.6) | 57.7 (50.7) | 55.7 (44.9) |
| Median (range), ng/ml |  | 45.8 (12.8, 126.9) | 48.5 (7.2, 233.7) | 47.6 (7.2, 233.7) |
|  |  |  |  |  |
| >10 years |  |  |  |  |
| N |  | 49 | 0 | 49 |
| Mean (SD), ng/ml |  | 45.2 (29.2) |  | 45.2 (29.2) |
| Median (range), ng/ml |  | 42.3 (0, 141.5) |  | 42.3 (0, 141.5) |
|  |  |  |  |  |
| Missing |  |  |  |  |
| N |  | 7 | 2 | 9 |
| Mean (SD), ng/ml |  | 59.7 (40.4) | 112.9 (58.7) | 71.5 (47.0) |
| Median (range), ng/ml |  | 41.9 (14.6, 118.1) | 112.9 (71.4, 154.4) | 56.7 (14.6, 154.4) |

**Table S4. Galectin-4 expression according to disease duration:**

| Duration | Controls  (n=40) | Crohn’s disease (n=97) | Ulcerative colitis (n=71) | All IBD  (n=168) |
| --- | --- | --- | --- | --- |
| None |  |  |  |  |
| N | 23 |  |  |  |
| Mean (SD), ng/ml | 35.7 (58.0) |  |  |  |
| Median (range), ng/ml | 0 (0, 185.5) |  |  |  |
|  |  |  |  |  |
| <1 year |  |  |  |  |
| N |  | 7 | 7 | 14 |
| Mean (SD), ng/ml |  | 7.8 (18.7) | 83.8 (149.9) | 45.8 (109.9) |
| Median (range), ng/ml |  | 0 (0, 50.2) | 38.4 (0, 417) | 0.8 (0, 417) |
|  |  |  |  |  |
| 1-5 years |  |  |  |  |
| N |  | 13 | 22 | 35 |
| Mean (SD), ng/ml |  | 56.0 (95.8) | 19.9 (44.8) | 33.3 (69.2) |
| Median (range), ng/ml |  | 1.8 (0, 275.2) | 0 (0, 140.2) | 0 (0, 275.2) |
|  |  |  |  |  |
| 5-10 years |  |  |  |  |
| N |  | 17 | 40 | 57 |
| Mean (SD), ng/ml |  | 25.7 (58.7) | 293.8 (1312.4) | 213.9 (1102.7) |
| Median (range), ng/ml |  | 0 (0, 216.6) | 0 (0, 8015) | 0 (0, 8015) |
|  |  |  |  |  |
| >10 years |  |  |  |  |
| N |  | 45 | 0 | 45 |
| Mean (SD), ng/ml |  | 30.7 (70.2) |  | 30.7 (70.2) |
| Median (range), ng/ml |  | 0 (0, 390.6) |  | 0 (0, 390.6) |
|  |  |  |  |  |
| Missing |  |  |  |  |
| N |  | 6 | 2 | 8 |
| Mean (SD), ng/ml |  | 228.9 (372.7) | 0.2 (0.2) | 171.7 (332.3) |
| Median (range), ng/ml |  | 0 (0, 868) | 0.2 (0, 0.3) | 0 (0, 868) |

**Table S5. Galectin-7 expression according to disease duration:**

| Duration | Controls  (n=40) | Crohn’s disease (n=97) | Ulcerative colitis (n=71) | All IBD  (n=168) |
| --- | --- | --- | --- | --- |
| None |  |  |  |  |
| N | 14 |  |  |  |
| Mean (SD), ng/ml | 132.2 (163.6) |  |  |  |
| Median (range), ng/ml | 60.1 (0, 466.3) |  |  |  |
|  |  |  |  |  |
| <1 year |  |  |  |  |
| N |  | 6 | 6 | 12 |
| Mean (SD), ng/ml |  | 53.2 (73.1) | 7.8 (19.2) | 30.5 (56.2) |
| Median (range), ng/ml |  | 16.1 (0, 170.6) | 0 (0, 47) | 0 (0, 170.6) |
|  |  |  |  |  |
| 1-5 years |  |  |  |  |
| N |  | 9 | 14 | 23 |
| Mean (SD), ng/ml |  | 179.6 (221.1) | 12.3 (18.9) | 77.8 (158.0) |
| Median (range), ng/ml |  | 111.2 (0, 572.4) | 0 (0, 50.9) | 12.1 (0, 572.4) |
|  |  |  |  |  |
| 5-10 years |  |  |  |  |
| N |  | 16 | 33 | 49 |
| Mean (SD), ng/ml |  | 24.4 (43.8) | 80.7 (195.4) | 62.3 (163.6) |
| Median (range), ng/ml |  | 0 (0, 147.3) | 0 (0, 859.7) | 0 (0, 859.7) |
|  |  |  |  |  |
| >10 years |  |  |  |  |
| N |  | 37 | 0 | 37 |
| Mean (SD), ng/ml |  | 48.4 (141.6) |  | 48.4 (141.6) |
| Median (range), ng/ml |  | 0 (0, 764.8) |  | 0 (0, 764.8) |
|  |  |  |  |  |
| Missing |  |  |  |  |
| N |  | 2 | 2 | 4 |
| Mean (SD), ng/ml |  | 15.2 (21.5) | 0 (0) | 7.6 (15.2) |
| Median (range), ng/ml |  | 15.2 (0, 30.4) | 0 (0, 0) | 0 (0, 30.4) |

**Table S6. Galectin-8 expression according to disease duration:**

| Duration | Controls  (n=40) | Crohn’s disease (n=97) | Ulcerative colitis (n=71) | All IBD  (n=168) |
| --- | --- | --- | --- | --- |
| None |  |  |  |  |
| N | 18 |  |  |  |
| Mean (SD), ng/ml | 19.4 (31.0) |  |  |  |
| Median (range), ng/ml | 4.7 (0, 104.2) |  |  |  |
|  |  |  |  |  |
| <1 year |  |  |  |  |
| N |  | 7 | 7 | 14 |
| Mean (SD), ng/ml |  | 95.5 (141.7) | 43.1 (45.5) | 69.3 (104.7) |
| Median (range), ng/ml |  | 7.5 (0, 334.9) | 30.7 (0, 128.3) | 25.2 (0, 334.9) |
|  |  |  |  |  |
| 1-5 years |  |  |  |  |
| N |  | 14 | 21 | 35 |
| Mean (SD), ng/ml |  | 68.9 (97.3) | 77.7 (96.7) | 74.2 (95.6) |
| Median (range), ng/ml |  | 25.0 (0, 287.1) | 34.4 (0, 320.7) | 34.4 (0, 320.7) |
|  |  |  |  |  |
| 5-10 years |  |  |  |  |
| N |  | 18 | 39 | 57 |
| Mean (SD), ng/ml |  | 82.2 (159.1) | 128.7 (410.4) | 114.0 (349.9) |
| Median (range), ng/ml |  | 1.7 (0, 578.5) | 7.0 (0, 2549.0) | 3.5 (0, 2549.0) |
|  |  |  |  |  |
| >10 years |  |  |  |  |
| N |  | 47 | 0 | 47 |
| Mean (SD), ng/ml |  | 20.9 (41.0) |  | 20.9 (41.0) |
| Median (range), ng/ml |  | 0 (0, 173.2) |  | 0 (0, 173.2) |
|  |  |  |  |  |
| Missing |  |  |  |  |
| N |  | 7 | 2 | 9 |
| Mean (SD), ng/ml |  | 56.1 (69.3) | 0 (0) | 43.7 (64.9) |
| Median (range), ng/ml |  | 35.3 (0, 199.4) | 0 (0, 0) | 20.8 (0, 199.4) |

**Table S7. Galectin-1 expression according to disease location:**

| Duration | Controls  (n=40) | Crohn’s disease (n=97) | Ulcerative colitis (n=71) | All IBD  (n=168) |
| --- | --- | --- | --- | --- |
| None |  |  |  |  |
| N | 15 |  |  |  |
| Mean (SD), ng/ml | 6.2 (14.2) |  |  |  |
| Median (range), ng/ml | 0.7 (0, 48.1) |  |  |  |
|  |  |  |  |  |
| Proctitis |  |  |  |  |
| N |  |  | 13 |  |
| Mean (SD), ng/ml |  |  | 13.4 (13.5) |  |
| Median (range), ng/ml |  |  | 10.1 (0.3, 48.9) |  |
|  |  |  |  |  |
| Left-sided |  |  |  |  |
| N |  |  | 32 |  |
| Mean (SD), ng/ml |  |  | 61.9 (167.0) |  |
| Median (range), ng/ml |  |  | 8.4 (0.2, 815.9) |  |
|  |  |  |  |  |
| Pancolitis |  |  |  |  |
| N |  |  | 16 |  |
| Mean (SD), ng/ml |  |  | 59.6 (189.1) |  |
| Median (range), ng/ml |  |  | 13.0 (0, 768.1) |  |
|  |  |  |  |  |
| Colonic |  |  |  |  |
| N |  | 21 |  |  |
| Mean (SD), ng/ml |  | 72.9 (261.2) |  |  |
| Median (range), ng/ml |  | 9.3 (0.9, 1210.1) |  |  |
|  |  |  |  |  |
| Ileal |  |  |  |  |
| N |  | 29 |  |  |
| Mean (SD), ng/ml |  | 58.0 (239.4) |  |  |
| Median (range), ng/ml |  | 7.5 (0.6, 1300) |  |  |
|  |  |  |  |  |
| Ileocolonic |  |  |  |  |
| N |  | 35 |  |  |
| Mean (SD), ng/ml |  | 10.1 (13.2) |  |  |
| Median (range), ng/ml |  | 5.4 (0.3, 66.0) |  |  |
|  |  |  |  |  |
| Perianal |  |  |  |  |
| N |  | 19 |  |  |
| Mean (SD), ng/ml |  | 77.2 (296.2) |  |  |
| Median (range), ng/ml |  | 7.7 (0.3, 1300) |  |  |

**Table S8. Galectin-2 expression according to disease location:**

| Duration | Controls  (n=40) | Crohn’s disease (n=97) | Ulcerative colitis (n=71) | All IBD  (n=168) |
| --- | --- | --- | --- | --- |
| None |  |  |  |  |
| N | 20 |  |  |  |
| Mean (SD), ng/ml | 177.7 (616.5) |  |  |  |
| Median (range), ng/ml | 0.6 (0, 2669.3) |  |  |  |
|  |  |  |  |  |
| Proctitis |  |  |  |  |
| N |  |  | 14 |  |
| Mean (SD), ng/ml |  |  | 1126.7 (3083.8) |  |
| Median (range), ng/ml |  |  | 0.03 (0, 11479.2) |  |
|  |  |  |  |  |
| Left-sided |  |  |  |  |
| N |  |  | 35 |  |
| Mean (SD), ng/ml |  |  | 3648.8 (16889.9) |  |
| Median (range), ng/ml |  |  | 0 (0, 99546.4) |  |
|  |  |  |  |  |
| Pancolitis |  |  |  |  |
| N |  |  | 17 |  |
| Mean (SD), ng/ml |  |  | 1928.4 (7164.2) |  |
| Median (range), ng/ml |  |  | 0 (0, 29589.9) |  |
|  |  |  |  |  |
| Colonic |  |  |  |  |
| N |  | 21 |  |  |
| Mean (SD), ng/ml |  | 224.6 (710.3) |  |  |
| Median (range), ng/ml |  | 0 (0, 3200.7) |  |  |
|  |  |  |  |  |
| Ileal |  |  |  |  |
| N |  | 32 |  |  |
| Mean (SD), ng/ml |  | 2250.2 (9953.3) |  |  |
| Median (range), ng/ml |  | 0.3 (0, 56371.4) |  |  |
|  |  |  |  |  |
| Ileocolonic |  |  |  |  |
| N |  | 38 |  |  |
| Mean (SD), ng/ml |  | 452.2 (2237.1) |  |  |
| Median (range), ng/ml |  | 0.03 (0, 13733.9) |  |  |
|  |  |  |  |  |
| Perianal |  |  |  |  |
| N |  | 20 |  |  |
| Mean (SD), ng/ml |  | 2994.0 (12569.4) |  |  |
| Median (range), ng/ml |  | 0 (0, 56371.4) |  |  |

**Table S9**. **Galectin-3 expression according to disease location:**

| Duration | Controls  (n=40) | Crohn’s disease (n=97) | Ulcerative colitis (n=71) | All IBD  (n=168) |
| --- | --- | --- | --- | --- |
| None |  |  |  |  |
| N | 23 |  |  |  |
| Mean (SD), ng/ml | 22.0 (22.1) |  |  |  |
| Median (range), ng/ml | 14.9 (0, 96.4) |  |  |  |
|  |  |  |  |  |
| Proctitis |  |  |  |  |
| N |  |  | 14 |  |
| Mean (SD), ng/ml |  |  | 50.3 (41.2) |  |
| Median (range), ng/ml |  |  | 32.7 (16.8, 167.7) |  |
|  |  |  |  |  |
| Left-sided |  |  |  |  |
| N |  |  | 36 |  |
| Mean (SD), ng/ml |  |  | 72.4 (63.8) |  |
| Median (range), ng/ml |  |  | 51.8 (9.8, 276.2) |  |
|  |  |  |  |  |
| Pancolitis |  |  |  |  |
| N |  |  | 20 |  |
| Mean (SD), ng/ml |  |  | 25.9 (21.6) |  |
| Median (range), ng/ml |  |  | 16.7 (0.7, 85.2) |  |
|  |  |  |  |  |
| Colonic |  |  |  |  |
| N |  | 25 |  |  |
| Mean (SD), ng/ml |  | 42.1 (28.8) |  |  |
| Median (range), ng/ml |  | 34.8 (0, 118.1) |  |  |
|  |  |  |  |  |
| Ileal |  |  |  |  |
| N |  | 33 |  |  |
| Mean (SD), ng/ml |  | 44.6 (50.1) |  |  |
| Median (range), ng/ml |  | 30.9 (0, 282.1) |  |  |
|  |  |  |  |  |
| Ileocolonic |  |  |  |  |
| N |  | 40 |  |  |
| Mean (SD), ng/ml |  | 61.3 (30.8) |  |  |
| Median (range), ng/ml |  | 55.5 (11.5, 141.5) |  |  |
|  |  |  |  |  |
| Perianal |  |  |  |  |
| N |  | 25 |  |  |
| Mean (SD), ng/ml |  | 53.0 (56.5) |  |  |
| Median (range), ng/ml |  | 40.1 (0, 282.1) |  |  |

**Table S10. Galectin-4 expression according to disease location:**

| Duration | Controls  (n=40) | Crohn’s disease (n=97) | Ulcerative colitis (n=71) | All IBD  (n=168) |
| --- | --- | --- | --- | --- |
| None |  |  |  |  |
| N | 23 |  |  |  |
| Mean (SD), ng/ml | 35.7 (58.0) |  |  |  |
| Median (range), ng/ml | 0 (0, 185.5) |  |  |  |
|  |  |  |  |  |
| Proctitis |  |  |  |  |
| N |  |  | 14 |  |
| Mean (SD), ng/ml |  |  | 20.1 (29.3) |  |
| Median (range), ng/ml |  |  | 0 (0, 78.2) |  |
|  |  |  |  |  |
| Left-sided |  |  |  |  |
| N |  |  | 37 |  |
| Mean (SD), ng/ml |  |  | 301.0 (1366.0) |  |
| Median (range), ng/ml |  |  | 0 (0, 8015) |  |
|  |  |  |  |  |
| Pancolitis |  |  |  |  |
| N |  |  | 20 |  |
| Mean (SD), ng/ml |  |  | 75.9 (108.2) |  |
| Median (range), ng/ml |  |  | 37.8 (0, 417) |  |
|  |  |  |  |  |
| Colonic |  |  |  |  |
| N |  | 25 |  |  |
| Mean (SD), ng/ml |  | 18.0 (55.7) |  |  |
| Median (range), ng/ml |  | 0 (0, 275.2) |  |  |
|  |  |  |  |  |
| Ileal |  |  |  |  |
| N |  | 33 |  |  |
| Mean (SD), ng/ml |  | 50.8 (91.9) |  |  |
| Median (range), ng/ml |  | 1.8 (0, 390.5) |  |  |
|  |  |  |  |  |
| Ileocolonic |  |  |  |  |
| N |  | 33 |  |  |
| Mean (SD), ng/ml |  | 17.7 (33.8) |  |  |
| Median (range), ng/ml |  | 0 (0, 125.2) |  |  |
|  |  |  |  |  |
| Perianal |  |  |  |  |
| N |  | 24 |  |  |
| Mean (SD), ng/ml |  | 93.5 (187.7) |  |  |
| Median (range), ng/ml |  | 3.5 (0, 868) |  |  |

**Table S11. Galectin-7 expression according to disease location:**

| Duration | Controls  (n=40) | Crohn’s disease (n=97) | Ulcerative colitis (n=71) | All IBD  (n=168) |
| --- | --- | --- | --- | --- |
| None |  |  |  |  |
| N | 14 |  |  |  |
| Mean (SD), ng/ml | 132.2 (163.6) |  |  |  |
| Median (range), ng/ml | 60.1 (0, 466.3) |  |  |  |
|  |  |  |  |  |
| Proctitis |  |  |  |  |
| N |  |  | 13 |  |
| Mean (SD), ng/ml |  |  | 50.8 (119.9) |  |
| Median (range), ng/ml |  |  | 0 (0, 439.4) |  |
|  |  |  |  |  |
| Left-sided |  |  |  |  |
| N |  |  | 28 |  |
| Mean (SD), ng/ml |  |  | 59.7 (191.9) |  |
| Median (range), ng/ml |  |  | 0 (0, 859.7) |  |
|  |  |  |  |  |
| Pancolitis |  |  |  |  |
| N |  |  | 13 |  |
| Mean (SD), ng/ml |  |  | 39.7 (100.5) |  |
| Median (range), ng/ml |  |  | 0 (0, 368.6) |  |
|  |  |  |  |  |
| Colonic |  |  |  |  |
| N |  | 19 |  |  |
| Mean (SD), ng/ml |  | 36.0 (98.8) |  |  |
| Median (range), ng/ml |  | 0 (0, 419.6) |  |  |
|  |  |  |  |  |
| Ileal |  |  |  |  |
| N |  | 22 |  |  |
| Mean (SD), ng/ml |  | 97.2 (175.5) |  |  |
| Median (range), ng/ml |  | 28.7 (0, 572.4) |  |  |
|  |  |  |  |  |
| Ileocolonic |  |  |  |  |
| N |  | 32 |  |  |
| Mean (SD), ng/ml |  | 66.7 (153.1) |  |  |
| Median (range), ng/ml |  | 0 (0, 764.8) |  |  |
|  |  |  |  |  |
| Perianal |  |  |  |  |
| N |  | 20 |  |  |
| Mean (SD), ng/ml |  | 79.2 (148.8) |  |  |
| Median (range), ng/ml |  | 6.1 (0, 548.6) |  |  |

**Table S12. Galectin-8 expression according to disease location:**

| Duration | Controls  (n=40) | Crohn’s disease (n=97) | Ulcerative colitis (n=71) | All IBD  (n=168) |
| --- | --- | --- | --- | --- |
| None |  |  |  |  |
| N | 18 |  |  |  |
| Mean (SD), ng/ml | 19.4 (31.0) |  |  |  |
| Median (range), ng/ml | 4.7 (0, 104.2) |  |  |  |
|  |  |  |  |  |
| Proctitis |  |  |  |  |
| N |  |  | 13 |  |
| Mean (SD), ng/ml |  |  | 54.5 (107.3) |  |
| Median (range), ng/ml |  |  | 8.8 (0, 382.9) |  |
|  |  |  |  |  |
| Left-sided |  |  |  |  |
| N |  |  | 36 |  |
| Mean (SD), ng/ml |  |  | 124.9 (425.5) |  |
| Median (range), ng/ml |  |  | 1.2 (0, 2549.0) |  |
|  |  |  |  |  |
| Pancolitis |  |  |  |  |
| N |  |  | 20 |  |
| Mean (SD), ng/ml |  |  | 101.1 (105.4) |  |
| Median (range), ng/ml |  |  | 70.3 (0, 382.9) |  |
|  |  |  |  |  |
| Colonic |  |  |  |  |
| N |  | 226 |  |  |
| Mean (SD), ng/ml |  | 64.9 (127.4) |  |  |
| Median (range), ng/ml |  | 11.9 (0, 578.5) |  |  |
|  |  |  |  |  |
| Ileal |  |  |  |  |
| N |  | 33 |  |  |
| Mean (SD), ng/ml |  | 47.2 (80.0) |  |  |
| Median (range), ng/ml |  | 7.5 (0, 287.1) |  |  |
|  |  |  |  |  |
| Ileocolonic |  |  |  |  |
| N |  | 37 |  |  |
| Mean (SD), ng/ml |  | 31.7 (81.2) |  |  |
| Median (range), ng/ml |  | 0 (0, 363.4) |  |  |
|  |  |  |  |  |
| Perianal |  |  |  |  |
| N |  | 24 |  |  |
| Mean (SD), ng/ml |  | 47.2 (90.1) |  |  |
| Median (range), ng/ml |  | 8.3 (0, 363.4) |  |  |

**Table S13. Galectin-1 expression according to medications:**

| Duration | Controls  (n=40) | Crohn’s disease (n=97) | Ulcerative colitis (n=71) | All IBD  (n=168) |
| --- | --- | --- | --- | --- |
| None |  |  |  |  |
| N | 15 |  |  |  |
| Mean (SD), ng/ml | 6.2 (14.2) |  |  |  |
| Median (range), ng/ml | 0.7 (0, 48.1) |  |  |  |
|  |  |  |  |  |
| 5-ASA |  |  |  |  |
| N |  | 19 | 49 | 68 |
| Mean (SD), ng/ml |  | 17.8 (23.1) | 61.3 (170.7) | 49.2 (146.3) |
| Median (range), ng/ml |  | 7.6 (1.4, 73.6) | 13.1 (0, 815.9) | 11.4 (0, 815.9) |
|  |  |  |  |  |
| Thiopurines |  |  |  |  |
| N |  | 35 | 16 | 51 |
| Mean (SD), ng/ml |  | 14.2 (17.5) | 130.8 (269.6) | 50.7 (158.1) |
| Median (range), ng/ml |  | 9.2 (0.3, 73.6) | 16.4 (0.7, 815.9) | 11.8 (0.3, 815.9) |
|  |  |  |  |  |
| Methotrexate |  |  |  |  |
| N |  | 2 | 0 | 2 |
| Mean (SD), ng/ml |  | 13.9 (16.3) |  | 13.9 (16.3) |
| Median (range), ng/ml |  | 13.9 (2.3, 25.4) |  | 13.9 (2.3, 25.4) |
|  |  |  |  |  |
| Anti-TNF agent |  |  |  |  |
| N |  | 15 | 3 | 18 |
| Mean (SD), ng/ml |  | 10.8 (9.6) | 12.6 (10.7) | 11.1 (9.5) |
| Median (range), ng/ml |  | 10.3 (0.3, 35) | 10.9 (2.8, 24) | 10.6 (0.3, 35) |
|  |  |  |  |  |
| Corticosteroids |  |  |  |  |
| N |  | 5 | 7 | 12 |
| Mean (SD), ng/ml |  | 5.3 (5.0) | 63.5 (114.8) | 39.3 (90.0) |
| Median (range), ng/ml |  | 4.9 (0.6, 13.3) | 16.7 (10.1, 322.9) | 13.2 (0.6, 322.9) |

**Table S14. Galectin-2 expression according to medications:**

| Duration | Controls  (n=40) | Crohn’s disease (n=97) | Ulcerative colitis (n=71) | All IBD  (n=168) |
| --- | --- | --- | --- | --- |
| None |  |  |  |  |
| N | 20 |  |  |  |
| Mean (SD), ng/ml | 177.7 (616.5) |  |  |  |
| Median (range), ng/ml | 0.6 (0, 2669.3) |  |  |  |
|  |  |  |  |  |
| 5-ASA |  |  |  |  |
| N |  | 23 | 53 | 76 |
| Mean (SD), ng/ml |  | 2654.9 (11729.6) | 3115.5 (14290.9) | 2976.1 (13490.8) |
| Median (range), ng/ml |  | 0.9 (0, 56371.4) | 0 (0, 99546.4) | 0.1 (0, 99546.4) |
|  |  |  |  |  |
| Thiopurines |  |  |  |  |
| N |  | 39 | 19 | 58 |
| Mean (SD), ng/ml |  | 64.2 (201.0) | 7615.2 (23421.8) | 2537.8 (13639.7) |
| Median (range), ng/ml |  | 0.2 (0, 888.4) | 0.5 (0, 99546.4) | 0.2 (0, 99546.4) |
|  |  |  |  |  |
| Methotrexate |  |  |  |  |
| N |  | 2 | 1 | 3 |
| Mean (SD), ng/ml |  | 0 (0) | 0 | 0 (0) |
| Median (range), ng/ml |  | 0 (0, 0) | 0 (0, 0) | 0 (0, 0) |
|  |  |  |  |  |
| Anti-TNF agent |  |  |  |  |
| N |  | 15 | 5 | 20 |
| Mean (SD), ng/ml |  | 65.2 (213.4) | 2760.3 (6168.1) | 739.0 (3078.4) |
| Median (range), ng/ml |  | 0 (0, 827) | 0.5 (0, 13794.1) | 0.03 (0, 13794.1) |
|  |  |  |  |  |
| Corticosteroids |  |  |  |  |
| N |  | 5 | 7 | 12 |
| Mean (SD), ng/ml |  | 177.8 (397.2) | 0.02 (0.06) | 74.1 (256.4) |
| Median (range), ng/ml |  | 0 (0, 888.4) | 0 (0, 0.02) | 0 (0, 888.4) |

**Table S15. Galectin-3 expression according to medications:**

| Duration | Controls  (n=40) | Crohn’s disease (n=97) | Ulcerative colitis (n=71) | All IBD  (n=168) |
| --- | --- | --- | --- | --- |
| None |  |  |  |  |
| N | 23 |  |  |  |
| Mean (SD), ng/ml | 22.0 (22.1) |  |  |  |
| Median (range), ng/ml | 14.9 (0, 96.4) |  |  |  |
|  |  |  |  |  |
| 5-ASA |  |  |  |  |
| N |  | 26 | 57 | 83 |
| Mean (SD), ng/ml |  | 64.1 (58.4) | 49.6 (53.3) | 54.1 (55.0) |
| Median (range), ng/ml |  | 44.9 (0, 282.1) | 27.5 (0.7, 276.2) | 32.7 (0, 282.1) |
|  |  |  |  |  |
| Thiopurines |  |  |  |  |
| N |  | 44 | 18 | 62 |
| Mean (SD), ng/ml |  | 47.7 (30.3) | 42.3 (30.6) | 46.1 (30.2) |
| Median (range), ng/ml |  | 42.7 (0, 141.5) | 28.6 (0.7, 128.9) | 42.1 (0, 141.5) |
|  |  |  |  |  |
| Methotrexate |  |  |  |  |
| N |  | 2 | 1 | 3 |
| Mean (SD), ng/ml |  | 58.6 (30.4) | 18.5 | 45.2 (31.6) |
| Median (range), ng/ml |  | 58.6 (37.1, 80.1) | 18.5 (18.5, 18.5) | 37.1 (18.5, 80.1) |
|  |  |  |  |  |
| Anti-TNF agent |  |  |  |  |
| N |  | 19 | 5 | 24 |
| Mean (SD), ng/ml |  | 50.8 (36.0) | 42.8 (33.9) | 49.1 (35.0) |
| Median (range), ng/ml |  | 48.6 (0, 126.9) | 46.6 (0.7, 85.2) | 47.6 (0, 126.9) |
|  |  |  |  |  |
| Corticosteroids |  |  |  |  |
| N |  | 6 | 9 | 15 |
| Mean (SD), ng/ml |  | 66.2 (26.7) | 31.0 (20.6) | 45.1 (28.6) |
| Median (range), ng/ml |  | 65.8 (35.7, 92.9) | 23.3 (11.4, 67.1) | 44.3 (11.4, 92.9) |

**Table S16. Galectin-4 expression according to medications:**

| Duration | Controls  (n=40) | Crohn’s disease (n=97) | Ulcerative colitis (n=71) | All IBD  (n=168) |
| --- | --- | --- | --- | --- |
| None |  |  |  |  |
| N | 23 |  |  |  |
| Mean (SD), ng/ml | 35.7 (58.0) |  |  |  |
| Median (range), ng/ml | 0 (0, 185.5) |  |  |  |
|  |  |  |  |  |
| 5-ASA |  |  |  |  |
| N |  | 23 | 58 | 81 |
| Mean (SD), ng/ml |  | 17.3 (39.2) | 218.5 (1092.9) | 161.4 (927.3) |
| Median (range), ng/ml |  | 0 (0, 156.8) | 0 (0, 8015) | 0 (0, 8015) |
|  |  |  |  |  |
| Thiopurines |  |  |  |  |
| N |  | 38 | 19 | 57 |
| Mean (SD), ng/ml |  | 52.3 (151.2) | 585.1 (1886.6) | 229.9 (1106.1) |
| Median (range), ng/ml |  | 0 (0, 868) | 0 (0, 8015) | 0 (0, 8015) |
|  |  |  |  |  |
| Methotrexate |  |  |  |  |
| N |  | 2 | 1 | 3 |
| Mean (SD), ng/ml |  | 62.6 (88.5) | 15.5 | 46.9 (68.2) |
| Median (range), ng/ml |  | 62.6 (0, 125.2) | 15.5 (15.5, 15.5) | 15.5 (0, 125.2) |
|  |  |  |  |  |
| Anti-TNF agent |  |  |  |  |
| N |  | 17 | 5 | 22 |
| Mean (SD), ng/ml |  | 22.8 (58.7) | 539.9 (1094.1) | 140.3 (529.0) |
| Median (range), ng/ml |  | 0 (0, 216.6) | 49.3 (0, 2494.7) | 0 (0, 2494.7) |
|  |  |  |  |  |
| Corticosteroids |  |  |  |  |
| N |  | 5 | 9 | 14 |
| Mean (SD), ng/ml |  | 2.0 (3.7) | 97.0 (130.2) | 63.1 (112.6) |
| Median (range), ng/ml |  | 0 (0, 8.4) | 66.3 (0, 417) | 5.1 (0, 417) |

**Table S17. Galectin-7 expression according to medications:**

| Duration | Controls  (n=40) | Crohn’s disease (n=97) | Ulcerative colitis (n=71) | All IBD  (n=168) |
| --- | --- | --- | --- | --- |
| None |  |  |  |  |
| N | 14 |  |  |  |
| Mean (SD), ng/ml | 132.2 (163.6) |  |  |  |
| Median (range), ng/ml | 60.1 (0, 466.3) |  |  |  |
|  |  |  |  |  |
| 5-ASA |  |  |  |  |
| N |  | 19 | 43 | 62 |
| Mean (SD), ng/ml |  | 37.3 (124.8) | 42.5 (120.9) | 40.9 (121.1) |
| Median (range), ng/ml |  | 0 (0, 548.6) | 0 (0, 584.7) | 0 (0, 584.7) |
|  |  |  |  |  |
| Thiopurines |  |  |  |  |
| N |  | 29 | 14 | 43 |
| Mean (SD), ng/ml |  | 46.3 (85.2) | 36.7 (97.3) | 43.1 (88.3) |
| Median (range), ng/ml |  | 0 (0, 419.6) | 0 (0, 368.6) | 0 (0, 419.6) |
|  |  |  |  |  |
| Methotrexate |  |  |  |  |
| N |  | 1 | 0 | 1 |
| Mean (SD), ng/ml |  | 0 |  | 0 |
| Median (range), ng/ml |  | 0 (0, 0) |  | 0 (0, 0) |
|  |  |  |  |  |
| Anti-TNF agent |  |  |  |  |
| N |  | 13 | 3 | 16 |
| Mean (SD), ng/ml |  | 31.0 (53.0) | 9.5 (16.5) | 26.9 (48.6) |
| Median (range), ng/ml |  | 0 (0, 155.2) | 0 (0, 28.6) | 0 (0, 155.2) |
|  |  |  |  |  |
| Corticosteroids |  |  |  |  |
| N |  | 5 | 6 | 11 |
| Mean (SD), ng/ml |  | 9.5 (14.3) | 0 (0) | 4.3 (10.3) |
| Median (range), ng/ml |  | 0 (0, 32.2) | 0 (0, 0) | 0 (0, 32.2) |

**Table S18. Galectin-8 expression according to medications:**

| Duration | Controls  (n=40) | Crohn’s disease (n=97) | Ulcerative colitis (n=71) | All IBD  (n=168) |
| --- | --- | --- | --- | --- |
| None |  |  |  |  |
| N | 18 |  |  |  |
| Mean (SD), ng/ml | 19.4 (31.0) |  |  |  |
| Median (range), ng/ml | 4.7 (0, 104.2) |  |  |  |
|  |  |  |  |  |
| 5-ASA |  |  |  |  |
| N |  | 25 | 56 | 81 |
| Mean (SD), ng/ml | a | 61.9 (128.3) | 110.3 (345.1) | 95.4 (295.5) |
| Median (range), ng/ml |  | 7.5 (0, 578.5) | 25.2 (0, 2549.0) | 19.3 (0, 2549.0) |
|  |  |  |  |  |
| Thiopurines |  |  |  |  |
| N |  | 41 | 19 | 60 |
| Mean (SD), ng/ml |  | 55.1 (76.6) | 208.7 (573.9) | 103.8 (331.2) |
| Median (range), ng/ml |  | 25.5 (0, 287.1) | 57.3 (0, 2549.0) | 29.6 (0, 2549.0) |
|  |  |  |  |  |
| Methotrexate |  |  |  |  |
| N |  | 2 | 1 | 3 |
| Mean (SD), ng/ml |  | 0 (0) | 67.9 | 22.6 (39.2) |
| Median (range), ng/ml |  | 0 (0, 0) | 67.9 (67.9, 67.9) | 0 (0, 67.9) |
|  |  |  |  |  |
| Anti-TNF agent |  |  |  |  |
| N |  | 18 | 5 | 23 |
| Mean (SD), ng/ml |  | 45.6 (84.1) | 587.5 (1104.4) | 163.4 (528.6) |
| Median (range), ng/ml |  | 6.6 (0, 334.9) | 67.9 (0, 2549.0) | 9.0 (0, 2549.0) |
|  |  |  |  |  |
| Corticosteroids |  |  |  |  |
| N |  | 5 | 9 | 14 |
| Mean (SD), ng/ml |  | 1.5 (3.4) | 59.5 (56.6) | 38.8 (52.9) |
| Median (range), ng/ml |  | 0 (0, 7.5) | 56.6 (0, 157.5) | 8.2 (0, 157.5) |

**Table S19. Galectin-1 expression according to disease severity:**

| Severity | Controls  (n=40) | Crohn’s disease (n=97) | Ulcerative colitis (n=71) |
| --- | --- | --- | --- |
| None |  |  |  |
| N | 15 |  |  |
| Mean (SD), ng/ml | 6.2 (14.2) |  |  |
| Median (range), ng/ml | 0.7 (0, 48.1) |  |  |
|  |  |  |  |
| Remission |  |  |  |
| N |  | 56 | 25 |
| Mean (SD), ng/ml |  | 55.5 (233.5) | 104.9 (232.5) |
| Median (range), ng/ml |  | 7.5 (0.3, 1300) | 13.1 (0.2, 815.9) |
|  |  |  |  |
| Mild |  |  |  |
| N |  | 10 | 20 |
| Mean (SD), ng/ml |  | 14.3 (20.5) | 12.9 (13.7) |
| Median (range), ng/ml |  | 5.5 (0.6, 66.0) | 8.4 (0, 48.9) |
|  |  |  |  |
| Moderate |  |  |  |
| N |  | 13 | 15 |
| Mean (SD), ng/ml |  | 15.3 (16.3) | 17.5 (15.6) |
| Median (range), ng/ml |  | 11.8 (0.8, 64.3) | 11.9 (1.1, 46.6) |
|  |  |  |  |
| Severe |  |  |  |
| N |  | 1 | 1 |
| Mean (SD), ng/ml |  | 7.1 | 10.9 |
| Median (range), ng/ml |  | 7.1 | 10.9 |
|  |  |  |  |
| Missing |  |  |  |
| N |  | 1 |  |
| Mean (SD), ng/ml |  | 5.8 |  |
| Median (range), ng/ml |  | 5.8 |  |
|  |  |  |  |

**Table S20. Galectin-2 expression according to disease severity:**

| Severity | Controls  (n=40) | Crohn’s disease (n=97) | Ulcerative colitis (n=71) |
| --- | --- | --- | --- |
| None |  |  |  |
| N | 20 |  |  |
| Mean (SD), ng/ml | 177.7 (616.5) |  |  |
| Median (range), ng/ml | 0.6 (0, 2669.3) |  |  |
|  |  |  |  |
| Remission |  |  |  |
| N |  | 59 | 25 |
| Mean (SD), ng/ml |  | 421.8 (1845.8) | 2078.7 (6474.6) |
| Median (range), ng/ml |  | 0.2 (0, 13733.9) | 0.1 (0, 29589.9) |
|  |  |  |  |
| Mild |  |  |  |
| N |  | 12 | 23 |
| Mean (SD), ng/ml |  | 5063.2 (16204.6) | 702.1 (2432.5) |
| Median (range), ng/ml |  | 0.5 (0, 56371.4) | 0 (0, 11479.2) |
|  |  |  |  |
| Moderate |  |  |  |
| N |  | 14 | 17 |
| Mean (SD), ng/ml |  | 520.9 (1489.4) | 6198.5 (24074.6) |
| Median (range), ng/ml |  | 0 (0, 5545.4) | 0 (0, 99546.4) |
|  |  |  |  |
| Severe |  |  |  |
| N |  | 1 | 1 |
| Mean (SD), ng/ml |  | 0.1 | 0 |
| Median (range), ng/ml |  | 0.1 | 0 |
|  |  |  |  |
| Missing |  |  |  |
| N |  | 1 |  |
| Mean (SD), ng/ml |  | 0 |  |
| Median (range), ng/ml |  | 0 |  |
|  |  |  |  |

**Table S21. Galectin-3 expression according to disease severity:**

| Severity | Controls  (n=40) | Crohn’s disease (n=97) | Ulcerative colitis (n=71) |
| --- | --- | --- | --- |
| None |  |  |  |
| N | 23 |  |  |
| Mean (SD), ng/ml | 22.0 (22.1) |  |  |
| Median (range), ng/ml | 14.9 (0, 96.4) |  |  |
|  |  |  |  |
| Remission |  |  |  |
| N |  | 63 | 30 |
| Mean (SD), ng/ml |  | 43.6 (30.2) | 46.3 (43.1) |
| Median (range), ng/ml |  | 40.6 (0, 141.5) | 28.3 (7.2, 186.4) |
|  |  |  |  |
| Mild |  |  |  |
| N |  | 16 | 23 |
| Mean (SD), ng/ml |  | 66.8 (65.3) | 55.6 (45.4) |
| Median (range), ng/ml |  | 53.4 (0, 282.1) | 47.7 (0.7, 167.7) |
|  |  |  |  |
| Moderate |  |  |  |
| N |  | 15 | 16 |
| Mean (SD), ng/ml |  | 50.9 (28.6) | 69.8 (78.1) |
| Median (range), ng/ml |  | 44.6 (12.8, 113.8) | 45.4 (13, 276.2) |
|  |  |  |  |
| Severe |  |  |  |
| N |  | 1 | 1 |
| Mean (SD), ng/ml |  | 24.1 | 85.2 |
| Median (range), ng/ml |  | 24.1 | 85.2 |
|  |  |  |  |
| Missing |  |  |  |
| N |  | 1 |  |
| Mean (SD), ng/ml |  | 110.2 |  |
| Median (range), ng/ml |  | 110.2 |  |
|  |  |  |  |

**Table S22. Galectin-4 expression according to disease severity:**

| Severity | Controls  (n=40) | Crohn’s disease (n=97) | Ulcerative colitis (n=71) |
| --- | --- | --- | --- |
| None |  |  |  |
| N | 23 |  |  |
| Mean (SD), ng/ml | 35.7 (58.0) |  |  |
| Median (range), ng/ml | 0 (0, 185.5) |  |  |
|  |  |  |  |
| Remission |  |  |  |
| N |  | 60 | 30 |
| Mean (SD), ng/ml |  | 37.9 (87.2) | 122.4 (457.6) |
| Median (range), ng/ml |  | 0 (0, 505.5) | 0 (0, 2494.7) |
|  |  |  |  |
| Mild |  |  |  |
| N |  | 13 | 23 |
| Mean (SD), ng/ml |  | 91.5 (239.0) | 26.4 (44.4) |
| Median (range), ng/ml |  | 1.8 (0, 868) | 0 (0, 140.2) |
|  |  |  |  |
| Moderate |  |  |  |
| N |  | 13 | 17 |
| Mean (SD), ng/ml |  | 35.0 (107.9) | 497.1 (1937.8) |
| Median (range), ng/ml |  | 0 (0, 390.5) | 0 (0, 8015) |
|  |  |  |  |
| Severe |  |  |  |
| N |  | 1 | 1 |
| Mean (SD), ng/ml |  | 55.5 | 49.3 |
| Median (range), ng/ml |  | 55.5 | 49.3 |
|  |  |  |  |
| Missing |  |  |  |
| N |  | 1 |  |
| Mean (SD), ng/ml |  | 0 |  |
| Median (range), ng/ml |  | 0 |  |
|  |  |  |  |

**Table S23. Galectin-7 expression according to disease severity:**

| Severity | Controls  (n=40) | Crohn’s disease (n=97) | Ulcerative colitis (n=71) |
| --- | --- | --- | --- |
| None |  |  |  |
| N | 14 |  |  |
| Mean (SD), ng/ml | 132.2 (163.6) |  |  |
| Median (range), ng/ml | 60.1 (0, 466.3) |  |  |
|  |  |  |  |
| Remission |  |  |  |
| N |  | 50 | 21 |
| Mean (SD), ng/ml |  | 41.0 (113.2) | 97.7 (226.6) |
| Median (range), ng/ml |  | 0 (0, 764.8) | 0 (0, 859.7) |
|  |  |  |  |
| Mild |  |  |  |
| N |  | 8 | 19 |
| Mean (SD), ng/ml |  | 179.3 (240.5) | 36.7 (101.6) |
| Median (range), ng/ml |  | 64.0 (0, 572.4) | 0 (0, 439.4) |
|  |  |  |  |
| Moderate |  |  |  |
| N |  | 11 | 14 |
| Mean (SD), ng/ml |  | 60.1 (125.9) | 9.6 (20.5) |
| Median (range), ng/ml |  | 0 (0, 419.6) | 0 (0, 62.7) |
|  |  |  |  |
| Severe |  |  |  |
| N |  | 0 | 1 |
| Mean (SD), ng/ml |  |  | 0 |
| Median (range), ng/ml |  |  | 0 |
|  |  |  |  |
| Missing |  |  |  |
| N |  | 1 |  |
| Mean (SD), ng/ml |  | 0 |  |
| Median (range), ng/ml |  | 0 |  |
|  |  |  |  |

**Table S24. Galectin-8 expression according to disease severity:**

| Severity | Controls  (n=40) | Crohn’s disease (n=97) | Ulcerative colitis (n=71) |
| --- | --- | --- | --- |
| None |  |  |  |
| N | 18 |  |  |
| Mean (SD), ng/ml | 19.4 (31.0) |  |  |
| Median (range), ng/ml | 4.7 (0, 104.2) |  |  |
|  |  |  |  |
| Remission |  |  |  |
| N |  | 62 | 29 |
| Mean (SD), ng/ml |  | 50.3 (102.6) | 134.7 (468.5) |
| Median (range), ng/ml |  | 3.6 (0, 578.5) | 19.7 (0, 2549.0) |
|  |  |  |  |
| Mild |  |  |  |
| N |  | 14 | 22 |
| Mean (SD), ng/ml |  | 73.0 (107.0) | 84.4 (116.0) |
| Median (range), ng/ml |  | 17.8 (0, 287.1) | 18.7 (0, 382.9) |
|  |  |  |  |
| Moderate |  |  |  |
| N |  | 15 | 17 |
| Mean (SD), ng/ml |  | 23.4 (56.0) | 69.8 (108.8) |
| Median (range), ng/ml |  | 0 (0, 199.4) | 0 (0, 403.5) |
|  |  |  |  |
| Severe |  |  |  |
| N |  | 1 | 1 |
| Mean (SD), ng/ml |  | 0 | 0 |
| Median (range), ng/ml |  | 0 | 0 |
|  |  |  |  |
| Missing |  |  |  |
| N |  | 1 |  |
| Mean (SD), ng/ml |  | 0 |  |
| Median (range), ng/ml |  | 0 |  |
|  |  |  |  |
