# Supplementary material for: Serum galectins as potential biomarkers of inflammatory bowel diseases
Source: PLoS One. 2020 Jan 13;15(1):e0227306. doi: 10.1371/journal.pone.0227306 (PMC6957193; doi:10.1371/journal.pone.0227306)
Supplement: Attachment — Submitted filename: Reviewer Recommendation and Comments for Manuscript Number PONE-D-19-23084R2.pdf [file pone.0227306.s004.pdf]

Reviewer Recommendation and Comments for Manuscript Number **PONE-D-19-23084R2**

Manuscript Title: SERUM GALECTINS AS POTENTIAL BIOMARKERS OF INFLAMMATORY BOWEL DISEASES

To Academic Editor of PLOS ONE

Dear Prof. Roger Chammas,

This current version is appropriate to be published. The second revision improved the quality of manuscript and contemplated an extensive analysis of data regarding to circulating galectins and biomarkers of IBD. Possible correlation between galectins and inflammatory bowel diseases (IBD), such as Crohn's disease and ulcerative colitis, is extremely relevant. Moreover, the potential to use galectin levels as biomarkers was clearly demonstrated in this manuscript. Given that authors satisfactorily responded the reviewer's comments, current format is recommended to publication.

Best regards

Felipe Leite de Oliveira
