# Supplementary material for: Serum galectins as potential biomarkers of inflammatory bowel diseases
Source: PLoS One. 2020 Jan 13;15(1):e0227306. doi: 10.1371/journal.pone.0227306 (PMC6957193; doi:10.1371/journal.pone.0227306)
Supplement: Attachment — Submitted filename: Sree-IBD MS-response to reviewers comments.docx [file pone.0227306.s002.docx]

October 22, 2019

Dr. Roger Chammas,

Academic Editor

PLOS ONE

Dear Dr. Chammas,

Thanks for sending us the reviewers’ very helpful comments. We have made substantial revision of the manuscript (including the addition of 18 new tables as supplementary supporting materials and also addition of 11 new references) according to the referees’ suggestions and comments including the addition of 18 new tables in supplement materials. Taking the reviewers’ points in turns:

Journal requirements:

1. Please ensure that your manuscript meets PLOS ONE's style requirements, including those for file naming. The PLOS ONE style templates can be found at
   <http://www.journals.plos.org/plosone/s/file?id=wjVg/PLOSOne_formatting_sample_main_body.pdf> and <http://www.journals.plos.org/plosone/s/file?id=ba62/PLOSOne_formatting_sample_title_authors_affiliations.pdf>

We have carefully edited the manuscript in accordance to the style requirements of PLOS One

2. Please provide additional details regarding participant consent. In the ethics statement in the Methods and online submission information, please ensure that you have specified (1) whether consent was informed and (2) what type you obtained (for instance, written or verbal). If your study included minors, state whether you obtained consent from parents or guardians. If the need for consent was waived by the ethics committee, please include this information.

Written informed consent was obtained from all participants in this study and the study population was comprised of adults over the age of 18. We have included these statements in the Method section (page 7).

3. Thank you for stating the following in the Competing Interests section:
"TBY, SD, NM and JR declare no conflicts of interest.
SS has received speaker fee from MSD, Actavis, Abbvie, Dr Falk pharmaceuticals, Shire and received educational grant from MSD, Abbvie.
LGY is an Academic Editor of PLOS ONE"

Please confirm that this does not alter your adherence to all PLOS ONE policies on sharing data and materials, by including the following statement: "This does not alter our adherence to  PLOS ONE policies on sharing data and materials.” (as detailed online in our guide for authors <http://journals.plos.org/plosone/s/competing-interests>).  If there are restrictions on sharing of data and/or materials, please state these. Please note that we cannot proceed with consideration of your article until this information has been declared.

This has been revised according to the suggestions (p19)

4. Please include your updated Competing Interests statement in your cover letter; we will change the online submission form on your behalf.

Updated Competing Interest statement is included: TBY, SD, NM and JR declare no conflicts of interest. SS has received speaker fee from MSD, Actavis, Abbvie, Dr Falk pharmaceuticals, Shire and received educational grant from MSD, Abbvie. LGY is an Academic Editor of PLOS ONE. This does not alter our adherence to PLOS ONE policies on sharing data and materials.

Reviewer 1 comments:

I am concerned by the analysis that uses a CRP threshold of 5mg/dL. The reference that they cite to provide this actually provides data to support a threshold of 5mg/L. This does not negate the findings that serum levels of galectin-1 and -3 are increased in IBD patients but it does affect much of the data analysis.

They do not really describe the issue with these markers being elevated in IBD but not acting as markers of disease activity. Discussion should center around the possibilities that either it is only an early step in the inflammatory cascade or that treatment effect also could cause this to falsely rise (rather than be a marker of inflammation).

Thanks the reviewer for spotting the Unit typo which has been corrected in the revision (5mg/L)

As suggested, we have added the below paragraph to discuss the discovery that galectins are elevated in IBD but are not effective markers of disease activity. “Although further studies with larger sample size are needed to confirm this conclusion, it is possible that the increase in serum galectins might represent an early event in the inflammatory cascade and not subsequently rise further with increase of disease activity. The recent reports showing that several galectin members play key pathogenic roles in animal models of colitis [new ref 27] and galectin-3 caused macrophage activation in the induction phase of colitis in a dextran sodium sulphate animal model of colitis[new ref 28] are in line with this possibility” (p17).

Minor points include a few spelling errors and that much of the data provided in the introduction is not appropriately referenced.

All the spelling errors in the manuscript have been corrected and three new references (refs 1 to 3) have been added in the introduction (p4)

Reviewer #2: GENERAL COMMENTS:

INTRODUCTION

1. The first paragraph needs some references.

Several new references were added (refs 1-4, p4)

2. Some references about galectins and IBD were neglected in the manuscript, such as Sundblad et al., 2018 and Cibor et al., 2019. Please, explore this theme in the introduction section. Moreover, Cibor and colleagues concluded that serum galectin-3 (and Gal-9) should not be considered biomarkers of IBD. Please, make a discussion regarding to galectins as biomarkers of IBD using more recent papers.

These suggested papers have all been discussed in the study and references included

MATERIALS AND METHODS
1. Subtitle of the first paragraph is absent

A subtitle of “Materials” has been added in the revision (p7)

RESULTS
1. Table 1 was appropriately used as base to correlate galectins levels with disease activity and C-reactive protein. However, to amplify the spectrum of analyzed data, the authors should explore underestimated data, such as disease location, disease duration, and medications:

We have conducted substantial additional analysis of serum galectin levels in accordance to disease location, disease duration and medications and included the results as 18 supplemental tables (Tables S1 to S18). These analyses showed no significant differences in median serum galectin values of any of the galectin members- 1, -2, -3, -4, -7 and -8 when stratified by disease location, duration or medications. We have included the results of those new analysis as supplement materials (Table S1 to S18) and add a statement in the main text as “We also analysed the serum levels of galectins-1, -2, -3, -4, -7, and -8 according to disease location (Table S1-S6), disease duration (Table S7-S12) and concomitant medications (Table S13-S18). No significant differences in median galectin values across the variables was observed for any galectin members (Table S1 to S18). As the sample size in each sub-group in this study was small, future studies with larger sample numbers would help to clarify this conclusion” (p11)

1.1 - Galectin expression and synthesis vary throughout the small and large intestines. Then, disease location can be a relevant factor to be included in the analysis.

1.2 – Disease duration can also be important to study of biomarkers. Galectins have differential expression depending on stage of diseases, such as inflammation and fibrosis.
1.3 – Medications can alter the inflammatory and immunological responses and consequently the expression, synthesis and/or secretion of galectins. Medications can be critical modulators of biomarkers and it is impossible to be neglected in study of biomarkers.

All have been revised as discussed above

1.4 - Values of serum levels of galectins are missed without a concentration unit as a reference (e.g. ng/mL).

Values of the serum galectin levels was added (ng/ml, Tables 2-4)

2. Table 2 was excellent to detect serum levels of galectins, in special galectin-1 and -3. However, some information is missing:
2.1 - The insertion of concentration unit (possibly ng/mL) can improve to readers.
2.2 – In table legend, please, include the meaning of “Gal-1 conc”, “Gal-2 conc”, etc.
3. Table 3 was critical to compare active and inactive UC. Again, the insertion of concentration unit (possibly ng/mL) can improve to readers.

Galectin concentration unit (ng/ml) was added and the word “conc” was removed to make it clearer

4. Table 4 was important to compare active and inactive CD. To better comprehension:
4.1 – Insert the concentration unit (possibly ng/mL) in values of serum galectins;
4.2 – The third column (“All”) shows an asterisk (*). Please, describe the meaning of this symbol.

Concentration unit (ng/ml) was included and a footnote was added for the asterisk “One patient whose disease activity could not be accurately established was also included in this analysis”

5. Table 5 showed a comparison with different protocols of analysis using Gal-1 and Gal-3 independently combined with CRP.

5.1 – Describe the unit (possibly ng/mL);

Concentration unit (ng/ml) was added

5.2 – It was not clear the strategy to obtain the values (“mean” and “median”). Please, describe in the text or in materials and methods section

This has now been clarified in the methods section with the addition of the following sentence: Mean and median serum galectin levels were calculated to account for inter-individual variability in serum galectin levels (p8).

5. Table 6 showed a ROC analysis. However, the text subsequent (below of table) can be reorganized to explain the values (unit) and symbols (“>=” is equivalent to “≥”?).

We have now clarified this by the addition of the following to the footnote of the table: The numerical value for the combination of serum CRP and galectin was obtained by simply combining the actual values.

DISCUSSION
1. Selection criteria for using galectin-1, -2, -3, -4, -7 and -8 should be discussed here. Why other galectins were not investigated in IBD patients? At least, these arguments can be detailed in materials and methods section.

The selection of these galectin members was entirely due to the commercial availability of the detection agents (capture and detection anti-galectin antibodies and recombinant galectins) of the sandwich galectin ELISA. We have made this clearer in the Method section (p8)

2. The authors discussed about immunomodulatory roles of galectin-3 inducing pro-inflammatory signals (such as IL-6 dependent manner). These aspects can be explored with more details, including galectin-1 in the context. Several cytokines are associated with galectin-1 and galectin-3 levels in IBD. Imbalances between pro and anti-inflammatory responses seem relevant to insert both galectins as biomarkers.

As suggested, we have added three new paragraphs in the Discussion section to discuss the immunomodulatory roles of galectin-1 and -3 and the galectin-associated cytokines in IBD (p17-19) and included a few new references (refs 27-30).

3. There are several neglected works using galectins to study the pathogenesis and progression of IBD. The insertion in discussion section will improve the quality of this manuscript.

As suggested, we have discussed the few early studies about the role of galectin-1 and -3 in IBD pathogenesis and progression in the discussion section and included a few new references (ref 27 to 31)

We hope the addition of the new data and the substantial revision has satisfactorily addressed the reviewers’ comments and the manuscript is now in an acceptable form for publication by PLOS One.

Yours sincerely,

Sreedhar Subramanian
