# Supplementary material for: Serum galectins as potential biomarkers of inflammatory bowel diseases
Source: PLoS One. 2020 Jan 13;15(1):e0227306. doi: 10.1371/journal.pone.0227306 (PMC6957193; doi:10.1371/journal.pone.0227306)
Supplement: Attachment — Submitted filename: Sree-IBD-revision-2-Response to reviewers conments.docx [file pone.0227306.s003.docx]

Dear Dr. Chammas,

Thanks for giving us the opportunity to address the reviewers’ further comments to this manuscript. We have revised the manuscript in accordance to these comments and included six additional tables in the supplement materials. Take these comments in turns:

Reviewer 2:

*In the materials and methods section, there is a statement that "CRP>=5mg/L was used to define active disease" although I believe the clinical indices were actually used to define activity or inactivity.*

Clinical indices were indeed used to define disease activity. We however also used a CRP threshold of >=5mg/L to compare the diagnostic sensitivity of CRP compared to serum galectins in differentiation of active from inactive disease. To make this clearer, we have added below in the revision: ‘We investigated the ability of CRP to distinguish active disease from remission using a threshold of CRP≥5mg/L, as described previously’ in the method section (page 7). (please also see responses below).

*I'm a bit concerned about the methods to add CRP and galectin-1 or galectin-3 to create a composite score to analyze against active or inactive IBD as it might underestimate the potential finding regarding an improvement in sensitivity for active IBD if you simply use the dichotomous variable of "galectin-1 normal or abnormal" and similarly for galectin-3.*

We acknowledge the limitations of combining serum CRP to galectins-1 and -3 to produce a composite measure hence included the below in the discussion section to make it clearer the exploratory nature of this combination of analysis: “As an exploratory assessment for any indication of increased discrimination, a combination of CRP with galectins1 and 3 was further analysed but neither galectins-1 nor -3 were unable to provide additive sensitivity of CRP in distinguishing active disease from remission” (p17).

*With regard to the interesting finding that neither galectin-1 or galectin-3 associate with disease activity, it's not clear what the spectrum of disease activity (based on HBI and SCCAI) was. If there is a predominance of mildly active patients, that may explain this finding as patients with moderate-severe activity might be more likely to have higher serum biomarkers than patients in the inactive state.*

To address this comment, we have now performed additional analyses of serum galectins-1 and -3 across the spectrum of disease activity. We used previously defined thresholds of Harvey-Bradshaw index and simple clinical colitis activity index to define clinical severity. Both in CD and UC, there were approximately equal number of patients with mild and moderate disease activity respectively. Only one patient had severe disease. We have included the results of these analysis in six new tables in the supplemental materials (Tables S19-S24) and added below in in the results section: ‘We also analysed serum galectin levels according to the clinical severity of disease activity (remission, mild, moderate or severe). No association was observed between serum galectin levels and increasing disease severity” (p12).

We hope these revisions have satisfactorily addressed all the reviewers’ comments and the manuscript is now in an acceptable form for publication.

With many thanks,

Sreedhar Subramanian

On behalf of all co-authors
